# *In silico* analysis of alpha1-antitrypsin variants: The effects of a novel mutation

**DOI:** 10.1590/S1415-47572010005000089

**Published:** 2010-12-01

**Authors:** Sabri Denden, Nadia Leban, Donia Hayek, Jalel Knani, Jemni Ben Chibani, Amel Haj Khelil

**Affiliations:** 1 Biochemistry and Molecular Biology Laboratory, Faculty of Pharmacy, MonastirTunisia; 2 Pulmonology Department, CHU Tahar Sfar, MahdiaTunisia

**Keywords:** alpha1-antitrypsin, computational analysis, damaging mutation

## Abstract

Alpha1-antitrypsin (AAT) is a highly polymorphic protein with more than 120 variants that are classified as normal (normal protein secretion), deficient (reduced circulating AAT level caused by defective secretion) or null (no protein secretion). Alpha1-antitrypsin deficiency, one of the most common genetic disorders, predisposes adults to pulmonary emphysema and, to a lesser extent, chronic liver disease and cirrhosis. In this report, we provide additional sequence data for alpha1-antitrypsin based on the characterization of a novel variant detected in a 53-year-old heterozygous patient with chronic obstructive pulmonary disease. The mutation occurred on a PI*M2 base allele and was characterized by a T → C transition at nt 97 in exon II that led to the replacement of phenylalanine by leucine (F33L). Since the mutation was found in the heterozygous state with the expression of a normally secreted variant (PI*M1) it was not possible to assess the pattern of F33L secretion. However, computational analyses based on evolutionary, structural and functional information indicated a reduction of 23 Å ^3^ in the side chain volume and the creation of a cavity in the protein hydrophobic core that likely disturbed the tridimensional structure and folding of AAT. The accuracy of the *in silico* prediction was confirmed by testing known mutations.

Alpha1-antitrypsin (AAT) is the archetypal protein of the serine proteinase inhibitor (SERPIN) superfamily. AAT is synthesized in hepatocytes and macrophages and protects the lower respiratory tract from proteolytic degradation by neutrophil elastase (Perlino *et al.*, 1987). This protein is encoded by a highly polymorphic locus, PI (for Proteinase Inhibitor), that consists of seven exons dispersed over 12.2 kb on chromosome 14q31-32.3 (Li et *al.,* 1998). The two parental PI genes are codominantly expressed and dictate the AAT serum level (Brantly et *al.,* 1991). The most common PI allele is designated PI*M and has a gene frequency of 0.95. Homozygosity of the PI*M allele results in AAT plasma concentrations ranging from 150 to 350 mg/dL, which provides a sufficient protease/antiprotease balance to protect lung tissues. The most frequent deficient variants are PI*S (p.E264V) and PI*Z (p.E342L). PI*S produces approximately 60% of PI*M alpha1-antitrypsin and the PI*Z allele produces 10%-15% of normal AAT levels. Other rarer deficiency states arising from null alleles result in no detectable AAT protein (Crystal, 1990). The major clinical consequence of AAT deficiency (AATD) is a high risk of early onset panlobular emphysema and, to a lesser extent, chronic liver disease and cirrhosis (Mahadeva and Lomas, 1998). Current gene frequency data indicate that AATD is one of the most common inherited disorders worldwide (de Serres *et al.*, 2007).

In this report, we provide additional sequence data for AAT based on the characterization of a novel AAT mutation discovered in a Tunisian patient with chronic obstructive pulmonary disease (COPD).

A 53-year-old man diagnosed with pulmonary emphysema was enrolled in a targeted AATD screening program run by the Pneumology Department of Ibn Eljazzar Hospital in Kairouan (central Tunisia). Based on a previously described algorithm (Denden et *al.,* 2009), all patients with obstructive lung disease and an AAT level < 150 mg/dL (threshold value) attended at this unit are screened for AATD. In this case, the AAT plasma concentration was 132 mg/dL, as measured using an immune turbidimetric assay (Denden et *al.,* 2009). The patient, an ex-smoker with a history of 28 pack-years, presented the common symptoms of airway obstructive disease, *i.e.*, breathlessness, cough, wheeze and phlegm. These symptoms began at 50 years of age. During a two-year follow-up, the patient showed increasing dyspnea and weight loss. An intradermal skin test and the quantification of total IgE (22.5 IU/mL) indicated that there was no atopy. At his first presentation, chest radiographs showed hyperinflation with an anterior diaphragmatic angle of 107 degrees in lateral view and a retrosternal airspace of 5.2 cm. Decreased lung markings and bullae were localized in the lower zones. Chest computed axial tomography confirmed the emphysematous state of the lower lobe and the presence of pulmonary parenchymal destruction seen as large areas of hypolucency and the presence of bullae. Baseline lung function tests were consistent with the onset of severe COPD: the forced expiratory volume in 1 s (FEV1) was 28% of the predicted value and the FEV1/FVC (forced vital capacity) ratio was 70%. After bronchodilator inhalation, the FEV1 improved 4.8% relative to the baseline value. During the follow-up, lung function impairment, assigned by the annual decline in FEV1 (ΔFEV1), was 213 mL/year.

Blood samples were collected for analysis after obtaining the patients informed consent and the investigation was approved by the local Ethics Committees of the institutions involved. DNA was isolated from whole peripheral blood using a standard phenol-chloroform method. Genotyping by RFLP-PCR was used to detect the most prevalent AATD mutations (S and Z) (Denden *et al.*, 2009). This analysis does not report any S or Z AAT allele. Sequencing of the AAT gene coding regions as previously described (Denden *et al.*, 2009) revealed a novel point mutation at c.97 T → C (Figure 1). This substitution led to the replacement of phenylalanine by leucine (F33L). Since the mutation was found in the heterozygous state, the PCR product of exon II of the SERPINA1 gene was cloned to identify the allele with the mutated base. Cloning was done using a TOPO-TA cloning kit (Invitrogen Corporation, Carlsbad, CA). The sequencing of positive clones confirmed the presence of the c.97 T → C point mutation on the PI*M2 (c.302 G → A) allele (Figure 2).

Although the mutation occurred in the heterozygous state, mild AATD, usually observed in subjects carrying only one deficient allele, could explain the disease. Indeed, a meta-analysis of previous studies indicated an increase in the risk of COPD in PI*MZ heterozygotes (Hersh *et al.*, 2004). The serum AAT level in this patient (132 mg/dL) was within the normal AAT range of the Tunisian population [120-280 mg/dL] (Denden et *al.,* 2008). However, the patient's heterozygous state meant that the expression of a normal allele (PI*M1) could have masked any deficiency arising from the mutated allele. Additional investigations were needed to elucidate this deficiency but, unfortunately, none of the patient's family members was available for further study.

To circumvent this limitation, computational methods were used to assess whether the mutation was detrimental to the protein structure and/or function. Changes in the stability of the mutant protein compared to the wild type protein were predicted using the Auto-Mute server (Masso and Vaisman, 2008). SIFT (Sorting Intolerant from Tolerant) software (Ng and Henikoff, 2002) was used to predict tolerance to the causal non-synonymous SNP (nsSNP) on the basis of sequence conservation in the protein family. The PolyPhen bioinformatic algorithm (Ramensky *et al.*, 2002) was used to predict the influence of the mutation based on data derived from structural parameters, functional annotations and evolutionary information, and the HybridMeth algorithm (Capriotti *et al.*, 2006) was used to predict the effect of the nsSNP based on evolutionary information. The p.F33L mutant was predicted to be 1.41 kcal mol^-1^ less stable than the wild type protein (PDB ID: 1atu). SIFT software showed that the phenylalanine at position 33 was highly conserved in the protein family (score of 0.00), while the HybridMeth algorithm favored a disease-associated nsSNP with a reliability index of 7. PolyPhen predicted that the p.F33L mutation was possibly deleterious to AAT (PSIC score difference of 1.882). The PolyPhen decision tree indicated that substitution of the buried amino acid F by L at position 33 in the AAT-A-α-helix led to a reduction in side chain volume of 23 Å^3^ and the creation of a cavity. Cavities in the protein hydrophobic core are very likely to disturb the tridimensional structure and folding of AAT. *In silico* analysis with known mutations was used to assess the accuracy of the predictions and confirmed that the computational analyses based on evolutionary, structural and functional information were in agreement with previous findings *in vivo* and *in vitro* (Table 1). A neutral effect was predicted for mutations associated with a normal AAT level: the common AAT mutations PI*M2 (R101H) and PI*M3 (E376D) apparently had no effect on the protein (Table 1). Mutations associated with a low AAT level were predicted to be damaging: AATD, observed in the most prevalent deficiency variants PI*S (E264V) and PI*Z (E342L), was attributed to a change in hydrophobicity and charge at buried sites of the protein. The rare allele PI*M_wurzburg_ (E369S) showed the same abnormality as the F33L mutation, *i.e.*, a 23 Å^3^ reduction in side chain volume (Table 1).

**Figure 1:**
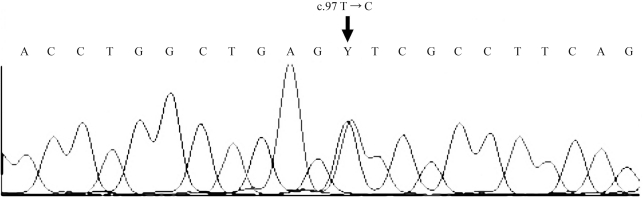
Partial exon II sequence of the *SERPINA1* gene (genomic DNA) reveals a novel c.97 T → C mutation at the heterozygous state.

**Figure 2:**
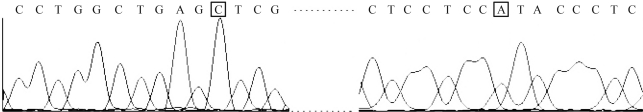
Partial exon II sequence of the *SERPINA1* gene (cloned DNA) reveals the presence of c.97 T → C and c.302 G → A substitutions on the same chromosome.

In conclusion, we have described a novel rare PI mutation that is likely to be clinically important, as suggested by computational analyses. Further studies are required to confirm this finding.

## Figures and Tables

**Table 1:** Computational analysis of alpha1-antitrypsin variants.

Mutation	Location	Auto-mute	SIFT	HybridMeth	PolyPhen		
		ΔΔG expected	Tolerance (score)	SPBP (RI)	PSIC difference	Substitution prediction	Substitution effect
Normally secreted variants							
R101H	hD	-0.29	Yes (0.47)	Neutral (6)	1.162	benign	NA
E376D	s4B	-0.17	Yes (0.56)	Neutral (7)	0.264	benign	Hydrophobicity change at buried site of 0.78; accN: 0.00
A34T	hA	-2.17	Yes (0.06)	Neutral (4)	0.657	benign	Distance to MET 385 of 3.345 Å
P88T	hD	-1.32	Yes (0.59)	Neutral (2)	0.937	benign	NA
A60T	hB-hC	-1.35	No (0.02)	Neutral (7)	1.001	benign	NA
G148R	s1A	-1.14	Yes (0.64)	Neutral (3)	0.631	benign	Charge change at exposed site, accN: 0.83
E204K	s4C	-0.89	Yes (0.98)	Neutral (3)	0.134	benign	NA
L276P	hH	-2.22	Yes (0.27)	Disease (5)	0.931	benign	Distance to ILE 375B of 3.807 Å
P362T	s1C	-1.45	Yes (0.52)	Neutral (8)	1.411	benign	NA
AAT Deficient variants							
E342L	s5A	0.87	No (0.00)	Disease (7)	3.190	probably damaging	Hydrophobicity change at buried site of 1.59, accN: 0.03; charge change at buried site
E264V	hG	0.32	No (0.03)	Disease (4)	1.773	probably damaging	Hydrophobicity change at buried site of 1.48; LYS 383H distance 2.658 Å; charge change at buried site, accN: 0.03
R223C	thIs5A	-1.18	No (0.04)	Disease (3)	0.671	possibly damaging	Cavity creation at buried site of -65 Å^3^; ligand: CIT distance 3.818 Å; hydrophobicity change at buried site of 1.66; charge change at buried site, accN: 0.15
L41P	hA	-1.67	No (0.00)	Disease (8)	2.621	probably damaging	Hydrophobicity change at buried site of 1.07, accN: 0.05
K335E	s5A	-0.17	No (0.00)	Disease (5)	1.612	possibly damaging	Distance to PHE 384B of 5.913 Å
M221T	s4C-s2B	-2.33	No (0.00)	Disease (6)	3.746	probably damaging	Hydrophobicity change at buried site of 1.02, accN: 0.00
T85M	hC-hD	0.07	Yes (0.09)	Disease (4)	2.400	probably damaging	Overpacking of 47 Å^3^, accN:0.90
R39C	hA	-0.86	No (0.00)	Disease (7)	2.872	probably damaging	Cavity creation at buried site of -65 Å^3^, accN: 0.35
P369S	s4B	-1.61	No (0.00)	Disease (3)	1.726	possibly damaging	Cavity creation at buried site of -23 Å^3^, accN: 0.20
P369L	s4B	-1.65	No (0.00)	Disease (5)	3.230	probably damaging	Overpacking of 55 Å^3^, accN: 0.20
D256V	ts2Bs3B-hG	0.53	Yes (0.11)	Disease (5)	1.698	possibly damaging	Distance to functional site SER 232A of 5.173 Å; distance to LYS 368B of 3. 307Å
G67E	hB-hC	-1.02	No (0.00)	Disease (8)	2.760	probably damaging	Overpacking at buried site of 78 Å^3^; hydrophobicity change at buried site of 2.44; Charge change at buried site, accN: 0.00
A336T	s5A	-0.26	Yes (0.08)	Disease (2)	0.993	probably damaging	Hydrophobicity change at buried site of 1.08; distance to PHE 384B of 4.320 Å, accN: 0.00
G225R	s4C-s2B	-0.48	Yes (0.11)	Disease (2)	0.714	probably damaging	Overpacking at buried site of 133 Å^3^; PRO 361B distance 5.919Å; hydrophobicity change at buried site of 3.3; charge change of a buried site, accN: 0.00
F33L	hA	-1.41	No (0.00)	Disease (7)	1.882	possibly damaging	Cavity creation at buried site of -23 Å^3^, accN: 0.00

ΔΔG expected: free energy change of protein stability at 25 °C, pH 7 (kcal mol^-1^) computed using the Auto-Mute server; Tolerance: tolerance to substitution scored using SIFT software; SPBP: sequence and profile based prediction of HybridMeth; RI: reliability index. PSIC difference: Position-Specific Independent Counts score difference between the two amino acids determined using the PolyPhen tool; accN: normed accessible surface area.
